# Mechanistic insights into pH-dependent structural and performance properties of diacylglycerol based high internal phase Pickering emulsion-Myofibrillar protein composite gels

**DOI:** 10.1016/j.fochx.2026.103896

**Published:** 2026-04-26

**Authors:** Ziyi Wang, Yuxin Huang, Yafei Zhou, Chao Zhang, Qian Liu, Qian Chen, Simin Zheng, Haotian Liu, Baohua Kong

**Affiliations:** a College of Food Science, Northeast Agricultural University, Harbin, Heilongjiang 150030, China; b DELISI GROUP Co., LTD. Delisi Industrial Park, Changcheng, Zhucheng, Weifang, Shandong, 262503, China

**Keywords:** Myofibrillar protein, Diacylglycerol, Soy protein isolate, Emulsion, Gel, pH

## Abstract

As a critical determinant, pH (5.5, 6.0, 6.5, 7.0) systematically influences the apparent properties and microstructure of composite gels comprising myofibrillar protein (MP) and diacylglycerol (DAG) based high internal phase Pickering emulsion (HIPPE). A marked decline in cooking, centrifugation, and thawing losses was observed with increasing pH, which underscored a pronounced enhancement in comprehensive stability, as evidenced by improved resistance to shear strain. The gel strength peaked at pH 6.0, beyond which a slight decrease was noted. Structural characterization revealed that higher pH conditions facilitated the formation of a denser, more continuous MP network and promoted a more uniform distribution of HIPPE droplets, with hydrophobic interactions and disulfide bonds identified as the dominant stabilizing forces. This work provides a theoretical foundation for quality improvement in meat products formulated with DAG HIPPE through controlled pH regulation.

## Highlights

• pH critically controls properties of diacylglycerol (DAG) based composite gels.
• Cooking, centrifugation, and thawing losses decrease with rising pH.
• Gel strength peaks at pH 6.0 and declines slightly at higher pH.
• Myofibrillar protein (MP) changes from aggregates to uniform network as pH increases.
• DAG phase droplet size decreases and distribution improves at elevated pH.

## Graphical abstract

**Figure:**
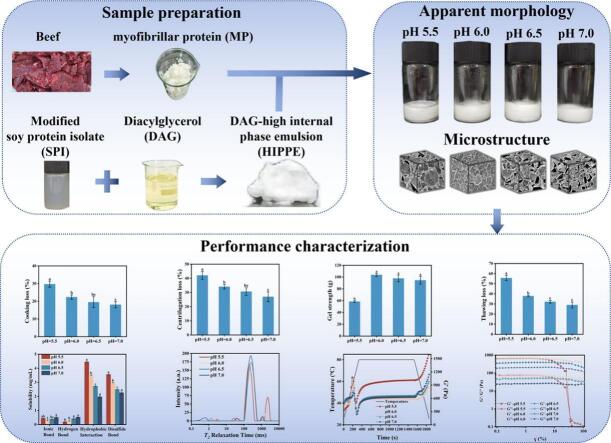

## 1 Introduction

Globally cherished for their distinctive textures, rich flavors, and succulence, meat products represent an essential component of culinary traditions. In recent years, however, growing health consciousness has shifted consumer expectations beyond mere sensory pleasure toward nutritional well-being (de Carvalho et al., 2025). While animal fat significantly contributes to the desirable organoleptic qualities of meat products, its excessive consumption has been associated with potential health risks, raising public concern (Wei et al., 2025). This has prompted extensive research into substituting animal fat with vegetable oils, which contain higher proportions of unsaturated fatty acids (Gumus-Bonacina et al., 2024). A key drawback of this substitution lies in the shared chemical composition of both substances. Like animal fats, vegetable oils are predominantly triacylglycerols (TAG), whose overconsumption is linked to obesity and cardiovascular disease (Christensen et al., 2024). In this context, diacylglycerol (DAG) has emerged as a promising alternative. As a natural component of edible oils, DAG possesses a unique molecular structure with one fewer fatty acid chain than TAG (Wang, Badar, et al., 2025). Its safety for consumption is well established, and accumulating clinical evidence highlights its distinct metabolic benefits, particularly the ability to suppress postprandial serum cholesterol and reduce body fat accumulation (Anikisetty et al., 2018; Devi et al., 2018). Nevertheless, a technical challenge arises from the liquid state of DAG, which contrasts with the solid nature of animal fat. Simply incorporating liquid DAG into meat batters tends to yield a loose, poorly cohesive product (Jiang & Xiong, 2015). To resolve this, pre emulsifying DAG into a high internal phase Pickering emulsion (HIPPE) has proven effective, successfully mitigating the structural deficiencies (Qi et al., 2025).

In our prior research, we developed a DAG HIPPE and effectively utilized it in beef patties as a full replacement for beef backfat (Badar, Wang, Zhou, et al., 2024). However, compared to traditional beef patties, those formulated with DAG HIPPE exhibited notably higher cooking loss and related quality defects (Badar, Wang, Zhou, et al., 2024). Overcoming these limitations is crucial to unlocking the full potential of DAG HIPPE in meat products. Such improvement demands a closer examination of the environmental and processing conditions that govern meat quality, among which pH often serves as a principal regulator of functional properties. For instance, phosphates commonly employed in meat processing act as effective buffers, altering the ionic milieu and maintaining pH stability (Molina et al., 2023). Conversely, the metabolic activity of microorganisms in fermented meat products drives a natural pH reduction (Fan et al., 2025). Additionally, sodium bicarbonate is often introduced to raise the final pH away from the isoelectric point of myofibrillar proteins (MPs), thereby improving water retention and tenderness (Li, Zou, et al., 2022). Together, these practical interventions affirm the central role of pH in directing the quality outcomes of processed meat products.

Critically, pH exerts its influence on meat product quality primarily through modifications to myofibrillar protein (MP) conformation and gelation behavior (Yan et al., 2024). As the most abundant and functionally crucial protein in muscle tissue, MP forms an extensive three-dimensional gel network upon heating, effectively entrapping water and fat molecules to impart desirable sensory characteristics (Yao, Huang, et al., 2025). This thermally induced gelation constitutes the fundamental process underlying meat texture development, where the resulting network architecture directly dictates the ultimate product quality. In composite systems such as DAG-HIPPE based patties, the MP gel matrix forms the continuous phase that provides the structural framework for the entire system, with its gelling properties playing a decisive role in determining final product performance (Wang, Zhou, et al., 2025). Thus, elucidating pH-dependent regulation of MP gelation represents an essential research direction for developing improved meat products where DAG HIPPE substitutes animal fat.

While the influence of pH on pure MP gels (Yang et al., 2020) or single emulsion systems (Wang, Miao, & Sun, 2025) has been individually documented, the underlying mechanisms remain poorly understood in complex multiphase systems such as DAG HIPPE-MP composites, which more closely mimic real meat products. A fundamental gap exists in explaining how pH acts as a master variable coordinating the structural evolution of both phases and translating MP-HIPPE interactions into macroscopic product quality. Currently, a systematic and cross-scale understanding of the synergistic and competitive dynamics between MP and HIPPE under pH regulation is still lacking.

Consequently, this research systematically examines pH-dependent changes in both performance properties and microstructural features of DAG HIPPE-MP composite gels. The composite gels were prepared across a pH gradient (5.5, 6.0, 6.5, 7.0). Their macroscopic performance was assessed through measurements of cooking loss, gel strength, water holding capacity (WHC), and thawing loss. The microstructural architecture was visualized using confocal laser scanning microscopy (CLSM) and cryo scanning electron microscopy (cryo-SEM). Furthermore, low field nuclear magnetic resonance (LF-NMR), molecular interaction analyses, and rheological measurements were employed to probe changes in the physicochemical structure of the gels. By integrating these multi-scale analyses, this work aims to establish a clear structure-property relationship for pH-dependent composite gels, thereby providing a theoretical framework for designing meat products wherein DAG HIPPE replaces animal fat.

## 2 Materials and methods

### 2.1 Materials

Beef striploins were sourced from Beidahuang Meat Corporation (Harbin, China). Diacylglycerol (DAG) derived from flaxseed oil (purity ≥99%) was obtained from Xi'an Xinlu Biotech Co., Ltd. (Xi'an, China), while soy protein isolate (SPI) with a protein content of ≥98% was supplied by Shandong Yuwang Biotechnology Co., Ltd. (Dezhou, China). All other chemicals and solvents used in the experiments were of analytical grade.

### 2.2 Preparation of DAG emulsion

The DAG emulsion was prepared following the protocol established in our prior publication (Badar, Wang, Sun, et al., 2024). In brief, 2 g of SPI was dissolved in 100 mL of deionized water and hydrated for 12 h at ambient temperature, followed by heating at 95 °C for 30 min under continuous magnetic stirring in a water bath. The resulting SPI dispersion was then subjected to ultrasonication at 450 W (23.10 W·cm^−2^) for 20 min using a 4.0 s on/4.0 s off pulsed mode at 20 kHz. Finally, the emulsion was formulated by combining flaxseed oil-derived DAG with the SPI dispersion at a 4:1 (*v*/v) ratio and homogenized at 13,000 rpm for 2.5 min using a T25 ULTRA-TURRAX® high-shear homogenizer (IKA-Werke GmbH & Co., Staufen, Germany). The emulsion was used within 6 h of preparation and stored at 4 °C prior to use.

### 2.3 Extraction of MPs

MPs were isolated according to the procedure reported by Yao, Liu, et al. (2025) with minor adaptations. The extraction process was initiated by mincing beef striploins free of visible fat and connective tissue, followed by homogenization (1:4, *w*/*v*) with 10 mM sodium phosphate buffer (PBS) containing 2 mM MgCl₂, 0.6 M NaCl, and 1 mM EGTA using a tissue crusher for 60 s. The resulting homogenate was subjected to centrifugation at 3500 ×*g* for 15 min in a Cence® L720R-3 refrigerated centrifuge maintained at 4 °C. After discarding the supernatant, the pellet was collected, and the extraction cycle was repeated twice. The accumulated pellets were subsequently resuspended in 0.6 M NaCl solution (1:4, w/v) and processed through three additional cycles under consistent conditions. Prior to the final centrifugation step, the dispersion pH was adjusted to 6.0 with 0.1 mol/L HCl and filtered through a four-layer gauze. Protein concentration was quantified by the biuret method using bovine serum albumin for calibration, yielding a protein purity of 13.72%–15.48% in the MP extract (calculated based on MP wet weight). All procedures were conducted at a controlled temperature of 4 °C in a refrigerated environment. Following extraction, MP was used promptly.

### 2.4 Preparation of DAG emulsion-MP composite gels

Four composite gel systems with varying pH levels were formulated by adjusting MP dispersions (40 mg/mL) in 0.6 M NaCl phosphate buffer (20 mM) to target pH values of 5.5, 6.0, 6.5, and 7.0. Each MP dispersion was combined with the prepared DAG emulsion at a fixed ratio of 4:1 (*w*/w). The mixtures were homogenized at 10,000 rpm for 60 s, then heated in an 80 °C water bath for 20 min. After heating, the samples were immediately cooled in an ice water bath and then allowed to return to room temperature before use. The resulting composite gels were designated as pH 5.5, pH 6.0, pH 6.5, and pH 7.0 according to their respective pH values. The composite gels were prepared in three separate batches to ensure reproducibility. Each batch was independently prepared following the identical protocol, and all subsequent analyses were performed on samples from each batch.

### 2.5 Cooking loss

Cooking loss was evaluated following the method described by Wang, Zhou, et al. (2025). Approximately 7 g of each composite gel sample was weighed into a 10 mL centrifuge tube, then heated in an 80 °C water bath for 20 min. After heating, the samples were cooled in an ice water bath and then allowed to return to room temperature. The sample was carefully blotted with filter paper to remove water that had formed during the heating process. The mass of the sample was recorded before and after the heating process. For each batch, four para llel samples were prepared per experimental group.

(1) $$\text{Cooking loss}\;\left(\%\right)=\frac{W_{1}-W_{2}}{W_{1}}\times 100$$

where W₁ and W₂ represent the weight of the gel before and after heating, respectively.

### 2.6 Centrifugation loss

Approximately 7 *g* of each gel sample was heated in a 10 mL centrifuge tube at 80 °C for 20 min, then cooled in an ice water bath and allowed to return to room temperature. Subsequently, the samples were centrifuged at 8000 *g* for 10 min at 4 °C. After centrifugation, the tubes were inverted onto filter paper to drain exuded water. Each batch included four parallel samples for every experimental group.

(2) $$\text{Centrifugation loss}\;\left(\%\right)=\frac{W_{3}-W_{4}}{W_{3}}\times 100$$

where W₃ and W₄ represent the sample weights before and after centrifugation, respectively.

### 2.7 Thawing loss

Thawing loss was evaluated according to a modified procedure adapted from Du et al. (2023). Gel samples (approximately 7 g) were first heated at 80 °C for 20 min, then cooled in an ice water bath and allowed to return to room temperature. The samples then underwent five freeze-thaw cycles, with each cycle consisting of 20 h at −20 °C followed by 4 h at 25 °C. Samples from each group within a batch were prepared in quadruplicate.

(3) $$\text{Thaw loss}\;\left(\%\right)=\frac{W_{5}-W_{6}}{W_{5}}\times 100$$

where W₅ and W₆ represent the sample weights before heating and after the fifth freeze-thaw cycle, respectively.

### 2.8 Gel strength

Gel strength was determined for the four composite gels using a modified version of the method reported by Yao, Huang, et al. (2025). Samples were loaded into small glass vials (2.7 cm in diameter) and filled to a height of approximately 3 cm. After filling, the vials were tapped gently on the bench top to remove air bubbles, ensuring a flat surface for subsequent measurement. For each experimental group within a batch, six parallel samples were prepared. Following thermal induction, the composite hydrogels were stored at 4 °C for 12 h, subsequently equilibrated at 25 °C for 2 h, and then subjected to testing. Measurements were performed with a TA-TXplusC texture analyzer equipped with a P/0.5 (12 mm) probe. The instrument was configured to compress each sample to 50% of its original height with the following parameters: a trigger force of 5 g, and pre-test, test, and post-test speeds all set at 2 mm/s.

### 2.9 LF-NMR analysis

Water distribution within the composite gels was characterized using LF-NMR according to the methodology established by Du et al. (2023). Approximately 5 g of the sample was carefully weighed and placed at the bottom of a 10 mL centrifuge tube. Subsequently, the composite gel was formed by heating and cooling. After ensuring the external surface of the tube was dry, the section of the tube containing the sample was positioned in the measurement cell for testing. For each group within a batch, samples were prepared in triplicate for measurement. Transverse relaxation time (*T₂*) was measured using a nuclear magnetic resonance imaging analyzer (MesoMR23-060H-I, Niumag Analytical Instrument Co., Ltd.). The measurements were performed with a spectral width of 200 kHz and a resonance frequency of 19 MHz.

### 2.10 CLSM analysis

The microstructure of the composite gels was examined using CLSM according to the methodology described by Badar, Wang, Zhou, et al. (2024). For staining, 0.5 mL of Nile Red and 0.4 mL of Nile Blue solutions were incorporated into 20 g of gel samples, followed by 30-min incubation in dark conditions. A small aliquot (approximately 0.02 g) of the stained gel was then mounted on a glass slide, covered with a coverslip, and immediately imaged. Fluorescence signals were captured at excitation wavelengths of 488 nm for Nile Red and 633 nm for Nile Blue, respectively. Observations were conducted using a confocal laser scanning microscope (Leica SP8, Leica Microsystems, Germany) equipped with a 40× dry objective lens.

### 2.11 Cryo-SEM analysis

Freshly prepared composite gels were mounted on copper supports and immediately immersed in liquid nitrogen for rapid vitrification. The frozen samples were then platinum-sputtered and transferred to the pre-cooled stage of a SU8010 cryo-SEM (Hitachi, Japan). Observation was conducted under the following conditions: stage temperature maintained at −140 °C and acceleration voltage set at 5 kV.

### 2.12 Molecular forces

Molecular forces within the composite gels were characterized following the procedure of Badar, Wang, Zhou, et al. (2024). In brief, 3 g gel samples were homogenized with 30 mL of four denaturing solutions: S1 (0.6 M NaCl), S2 (0.6 M NaCl +1.5 M urea), S3 (0.6 M NaCl +8 M urea), and S4 (0.6 M NaCl +8 M urea +0.5 M β-mercaptoethanol). The mixtures were then centrifuged at 10,000 *g* for 15 min at 4 °C. Protein content in the resulting supernatants was quantified by the biuret method using bovine serum albumin for calibration. The solubility differences among the four treatments were interpreted as representing contributions from ionic bonds (S1), hydrogen bonds (S2-S1), hydrophobic interactions (S3-S2), and disulfide bonds (S3-S4). Three replicate samples were prepared for each group in every batch.

### 2.13 Rheological properties

The rheological properties were determined using a HAAKE MARS60 modular rheometer (Thermo Fisher Scientific, China) equipped with 40 mm parallel plates. Measurements were conducted with a 1 mm gap. A 2 mL sample aliquot was used and the exposed edge was sealed with silicone oil to prevent evaporation.

#### 2.13.1 Temperature-time sweep test

Samples were subjected to the following thermal protocol heating from 25 to 80 °C at 10 °C/min, maintaining at 80 °C for 20 min, then cooling to 25 °C at 10 °C/min. Storage modulus G' and loss modulus G" were monitored throughout at an oscillation frequency of 10 rad/s and 1% strain.

#### 2.13.2 Shear strain test

The linear viscoelastic region LVR was determined by applying shear strains ranging from 0.1% to 100%.

#### 2.13.3 Frequency sweep test

Measurements were performed at a constant strain of 1% while scanning frequencies from 0.1 to 10 Hz.

### 2.14 Statistical analysis

Statistical analysis was conducted utilizing SPSS (v26, IBM) to assess the influence of pH on the quality characteristics of composite gels, including cooking loss, centrifugation loss, thawing loss, gel strength, LF-NMR analysis and molecular forces. Analysis of variance (ANOVA) was utilized, incorporating pH levels as fixed effects and experimental batches as random effects. Significant differences (*P* < 0.05) among treatment means were determined utilizing Duncan's multiple range test. Results are presented graphically as mean ± standard error, created using Origin 2021 software.

## 3 Results and discussion

### 3.1 Cooking loss

**Fig. 1:**
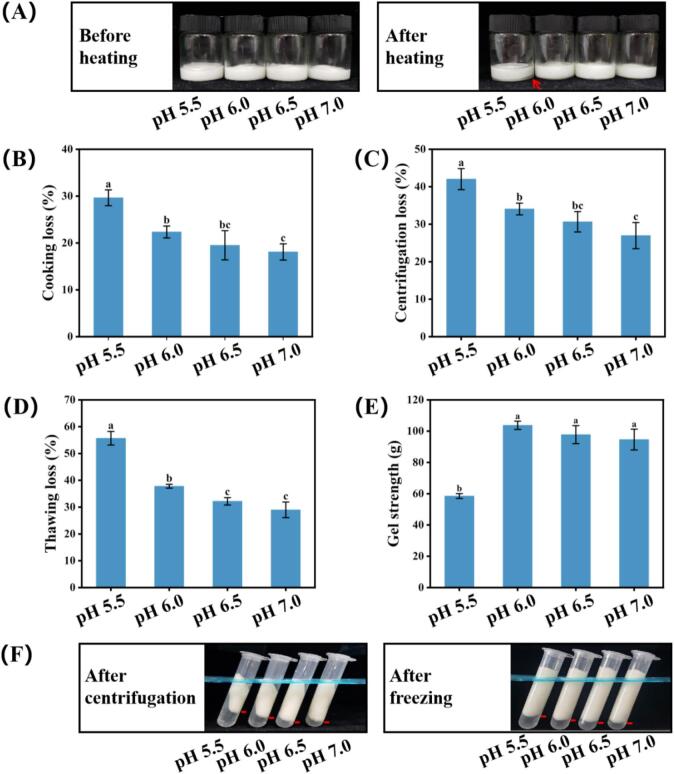
(A) Visual appearance before and after heating, (B) cooking loss, (C) centrifugation loss, (D) thawing loss, (E) gel strength, and (F) visual appearance after centrifugation and freeze-thaw cycling of composite gels at different pH levels (5.5, 6.0, 6.5, and 7.0). ^a-c^ Mean values with different superscripts indicate statistically significant differences (*P* < 0.05) among pH treatments.

Fig. 1A illustrates the composite gels before and after heating. The sample at pH 5.5 displayed marked phase separation following heating due to considerable water loss, whereas the other three pH groups maintained structural integrity with no visible separation. Fig. 1B summarizes the cooking loss results across different pH values. The highest cooking loss (29.64%) was observed at pH 5.5, while the lowest (18.09%) occurred at pH 7.0. A consistent downward trend in cooking loss was observed with increasing pH (*P* < 0.05), aligning with earlier findings in porcine MP gels (Hong GeunPyo et al., 2011). The enhanced water retention arises from synergistic improvements in both the MP gel matrix and SPI-stabilized HIPPE. Given that the isoelectric points of MP and SPI are approximately 5.5 and 4.5, respectively, the pH 5.5 condition situates the composite system near these critical points. This results in minimal net surface charge on both proteins, reducing their solubility (Lam & Nickerson, 2015). Under these conditions, MP fails to unfold completely, forming a loose and poorly hydrated gel network (Liu et al., 2021). SPI exhibits weak adsorption at the oil water interface due to its compact structure near the isoelectric point, which limits its emulsifying capacity and interfacial stability (Feng et al., 2025). The poor performance of both proteins collectively contributes to the high cooking loss at pH 5.5. As the pH increases, the net negative charge on both MP and SPI rises. The heightened electrostatic repulsion shifts MP from an aggregated state to a unfolded conformation, enhancing its solubility (Huang et al., 2024). This structural rearrangement allows MP to form a heat-induced gel with a strengthened three-dimensional network, improving its capacity to entrap water and other constituents, thereby reducing cooking loss. Similarly, the increased negative charge promotes structural unfolding in SPI, exposing more hydrophobic regions (Yamauchi et al., 1991). This enables SPI to form a dense, charged interfacial film around the DAG droplets, which not only stabilizes the HIPPE structure but also works synergistically with MP to minimize cooking loss in the composite gel.

### 3.2 Centrifugation loss

Centrifugation loss serves as an indicator of composite gel stability under external stress. As shown in Fig. 1C, the centrifugation loss was significantly influenced by pH, with the highest value (42.04%) observed at pH 5.5 and the lowest (26.96%) at pH 7.0. Correspondingly, the phase separation after centrifugation showed progressive improvement with increasing pH (Fig. 1F), following a trend consistent with the cooking loss results. This parallel behavior can be attributed to pH-induced modifications in both MP and SPI properties. At pH 5.5, the loose three-dimensional MP network failed to effectively immobilize the aqueous phase (Yang et al., 2020). During centrifugation, liquid phase accumulation disrupted the continuous MP gel matrix, leading to rapid leakage through porous structures and consequently high centrifugation loss. With increasing pH, MP exposed more sulfhydryl groups, promoting enhanced disulfide bond cross-linking during heating (Wang et al., 2022). This produced a more compact gel network with a significantly improved capacity to bind water. Concurrently, higher pH increased the net negative charge on SPI, enabling the formation of a dense interfacial film on DAG surfaces that reduced HIPPE droplet size (Li, Zhang, et al., 2024). The finer HIPPE droplets distributed more uniformly throughout the MP gel network, serving as functional fillers that created physical barriers against water migration. The refined MP network combined with the homogeneous HIPPE distribution collectively contributed to reduced centrifugation loss. These findings align with previous reports describing the beneficial effect of alkaline conditions on minimizing centrifugation loss in MP gels (Wang, Li, et al., 2025; Yang et al., 2020), while confirming that this principle remains valid in the more complex HIPPE-MP composite system.

### 3.3 Thawing loss

The thawing loss of the composite gels, which reflects their freeze-thaw stability under different pH conditions, is presented in Fig. 1D. The visual appearance of all treatment groups after five freeze-thaw cycles is shown in Fig. 1F. Thawing loss decreased significantly with increasing pH, from 55.70% at pH 5.5 to 28.99% at pH 7.0, following the same trend observed for cooking loss and centrifugation loss. The freeze-thaw process damages both the continuous MP phase and the dispersed HIPPE phase. At pH 5.5, the fragile three-dimensional MP network exhibited poor liquid binding capacity. During freezing, ice crystal growth mechanically damaged the already vulnerable MP matrix and the oil-water interface of HIPPE (Hu et al., 2025), exacerbating liquid loss upon thawing (Liu et al., 2016).

At elevated pH levels, the MP network became more compact and resistant to ice crystal damage, enabling better liquid retention. Meanwhile, HIPPE instability during freeze-thaw cycling primarily resulted from water phase freezing. Ice expansion generated stress that disrupted the HIPPE structure. Since the freezing point of the oil phase is lower than that of the water phase, water crystallization reduced the amount of liquid water, increasing the oil volume fraction and promoting droplet aggregation, thereby reducing HIPPE stability (Palazolo et al., 2011). The interfacial film formed by SPI on DAG surfaces was susceptible to disruption by water crystallization. At low temperatures, SPI tends to aggregate and distribute unevenly, further damaging the interfacial film. However, the SPI used in this study underwent heat treatment during HIPPE preparation. Compared to native SPI, heat-denatured SPI showed reduced aggregation tendency during freezing (Palazolo et al., 2011), thereby mitigating interfacial film damage. Increasing the pH away from the isoelectric point of SPI reduced SPI flocculation and enhanced its adsorption capacity to DAG, consequently minimizing droplet aggregation (McClements, 2004; Palazolo et al., 2011). Higher pH in the composite gels thus enhanced HIPPE droplet stability and promoted their distribution within an MP matrix with improved freeze-thaw resistance. This synergistic interaction between the dispersed and continuous phases collectively reduced the thawing loss of composite gels.

### 3.4 Gel strength

The gel strength of composite gels under different pH conditions is shown in Fig. 1E. As pH increased from 5.5 to 6.0, gel strength rose significantly from 58.52 g to 103.74 g. This substantial increase relates directly to the state of the MP network formation, where higher gel strength indicates greater cross-linking density (Chen et al., 2025). Variations in pH alter the hydration and electrostatic interactions between protein molecules, consequently affecting the physical integrity of the three-dimensional MP matrix (Liu et al., 2008; Yang et al., 2020). At pH 5.5, close to the isoelectric point of MP, the protein formed a weakly cross-linked and fragile network structure, resulting in minimal gel strength. When pH increased to 6.0, moving away from the isoelectric point, MP acquired more negative charges. The enhanced electrostatic repulsion promoted protein unfolding and orderly cross-linking, leading to the formation of a compact three-dimensional network (Du et al., 2022). However, when pH further increased to 6.5 and 7.0, the gel strength showed a slight, though statistically insignificant, decline. This may be because the increased electrostatic repulsion at higher pH weakens the interfacial anchoring between the SPI-stabilized oil droplets and the MP gel matrix, thereby reducing the synergistic reinforcement between the two phases. Moreover, when oil droplets become too small and densely distributed, they may interrupt the continuity of the MP gel phase, slightly compromise structural coherence and consequently reducing the gel strength (Yao, Huang, et al., 2025).

### 3.5 Lf-NMR

**Fig. 2:**
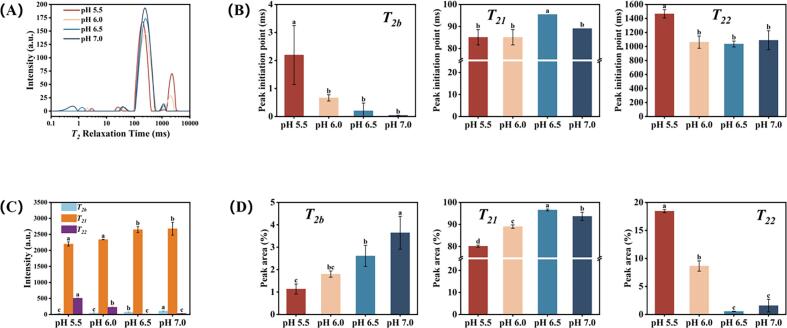
(A) *T*_*2*_ relaxation time curves, (B) peak relaxation time, (C) integrated signal amplitude, and (D) area proportion of various peaks in MP composite gel with different pH levels (5.5, 6.0, 6.5, and 7.0). *T*_*2b*_: the *T*_*2*_ relaxation time of bound water; *T*_*21*_: the *T*_*2*_ relaxation time of immobilized water; *T*_*22*_: the *T*_*2*_ relaxation time of free water. ^a-d^ Mean values with different superscripts are statistically different (*p* < 0.05) in different pH levels.

The water distribution within the composite gels, as determined by LF-NMR, is presented in Fig. 2. The *T*_*2*_ relaxation spectra exhibited three distinct peaks within the 0.1–10,000 ms range, corresponding to bound water (*T*_*2b*_, 1–40 ms), immobilized water (*T*_*21*_, 40–700 ms), and free water (*T*_*22*_, 700–4000 ms). With increasing pH, systematic shifts in peak initiation points were observed (Fig. 2A and B). The *T*_*2b*_ peak shifted toward shorter relaxation times, moving from 2.196 ms to 0.044 ms. The *T*_*21*_ peak demonstrated a more complex behavior, initially shifting from 85.091 ms (pH 5.5) to 95.477 ms (pH 6.5) before returning to 89.074 ms (pH 7.0). Concurrently, the *T*_*22*_ peak shifted leftward from 1465.762 ms to 1089.720 ms. Quantitative analysis revealed that immobilized water constituted the dominant fraction across all treatments, while both bound water and free water represented relatively minor components (Fig. 2C and D).

As pH increased, the proportions of both bound water and immobilized water showed an increasing trend, while free water progressively decreased. These changes in water binding states primarily reflect pH-induced modifications in MP conformation. Near the isoelectric point at pH 5.5, MP underwent significant aggregation, burying polar groups within the protein structure and limiting their availability for water binding (Matak et al., 2015). The resulting loose gel network consequently contained minimal bound water. With increasing pH, the elevated net negative charge on MP enhanced electrostatic repulsion, promoting myosin unfolding and exposing additional polar groups (Hu et al., 2025). This structural transformation significantly improved water binding capacity, leading to increased bound water content and the observed leftward shift in its peak initiation point. Furthermore, the expanded MP conformation at higher pH facilitated the formation of a more compact gel matrix. The finer network structure restricted water mobility, while simultaneously, pH elevation promoted the generation of smaller HIPPE droplets (Li, Cao, et al., 2025). These droplets uniformly distributed within the MP network pores, creating physical barriers that further impeded water migration, collectively contributing to the rising immobilized water fraction. The maximum immobilized water content (96.57%) occurred at pH 6.5.

In contrast to the consistent trends observed for bound and free water, the immobilized water fraction exhibited a distinctive rightward shift in peak initiation point. Similar patterns have been reported in previous studies (Bertram et al., 2004; Yang et al., 2020). We hypothesize that this phenomenon reflects water reorganization within the composite gel matrix. At pH 6.5, both the HIPPE droplets and previously unbound water molecules from lower pH systems became more effectively incorporated into the MP network. This enhanced entrapment increased the immobilized water proportion, while the incorporation of additional water molecules simultaneously caused the observed rightward migration of its peak initiation point. At pH 7.0, the strengthened water-binding capacity of the MP network led to a leftward shift in the immobilized water peak, though the relaxation time remained higher than those observed at pH 5.5 and 6.0. The water distribution patterns align consistently with the cooking loss (Fig. 1B), centrifugation loss (Fig. 1C), and thawing loss (Fig. 1D) results. Higher proportions of bound and immobilized water, coupled with reduced free water content, directly correlate with improved gel stability and correspondingly lower water losses during processing and storage.

### 3.6 CLSM

**Fig. 3:**
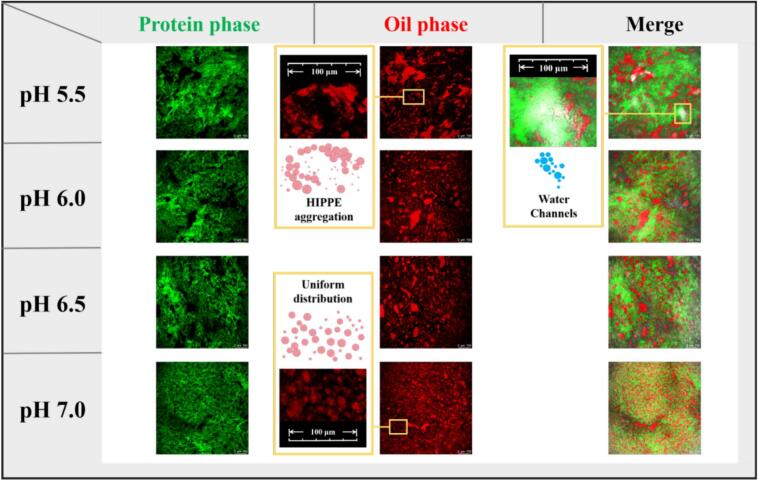
CLSM images of composite gel with different pH levels (5.5, 6.0, 6.5, and 7.0).

Fig. 3 presents the CLSM images of the composite gels, where green areas represent the continuous MP network and red regions indicate the dispersed HIPPE phase encapsulated within the protein matrix. Heat treatment promotes myosin unfolding and subsequent cross-linking, leading to the development of a continuous three-dimensional network (Liu et al., 2021) that effectively entraps and immobilizes the HIPPE droplets. At pH 5.5, the composite gel displayed a heterogeneous structure with alternating dense and loose regions. Within the green MP continuous phase, significant protein aggregation occurred instead of proper network formation, resulting in an uneven structural distribution. This microstructural inhomogeneity can be attributed to the pH proximity to the MP isoelectric point, where insufficient electrostatic repulsion (Huang et al., 2024), combined with exposed hydrophobic residues during thermal denaturation, amplified intermolecular hydrophobic interactions and promoted large aggregate formation. The defective continuous phase consequently led to poor dispersion of the red HIPPE droplets. Both oil droplets and water channels (white areas) accumulated in interstitial spaces not occupied by the MP network, creating extensive regions of oil and water aggregation. These water channels formed because the loose and discontinuous MP network at pH 5.5 lacked sufficient structural integrity to uniformly retain the aqueous phase. This allowed free water to coalesce into visible channels, which further impaired gel stability by serving as pathways for liquid migration and structural collapse.

Furthermore, the interfacial protein film enveloping larger oil droplets contained substantial protein-to-protein voids. During heating, oil droplet expansion caused partial release of lipid phase through these interfacial defects (Gordon et al., 1992), manifesting as fine red oil phases surrounding larger droplets in CLSM images. As pH increased beyond the MP isoelectric point, enhanced electrostatic repulsion worked synergistically with thermal treatment to suppress MP aggregation, promoting the formation of a homogeneous and densely organized three-dimensional gel network. This structural improvement simultaneously mitigated HIPPE aggregation. Consequently, in these higher pH systems, the water phase was more effectively integrated into the gel matrix, with no visible water channel formation, indicating superior water retention and structural uniformity. Concurrently, the elevated net negative charge on SPI strengthened intermolecular electrostatic repulsion, facilitating superior adsorption at the oil-water interface and generating smaller, more stable oil droplets (Li & Yu, 2023). The densely organized MP matrix physically restricted HIPPE mobility and aggregation, maintaining reduced droplet size. However, the extremely compact network structure and finely dispersed oil droplets may have restricted local chain mobility within the continuous phase, potentially compromising its resistance to mechanical stress (Yao, Huang, et al., 2025). The slight decrease in gel strength observed between pH 6.0 and 7.0 (Fig. 1E) can be explained by these microstructural transformations.

### 3.7 Cryo-SEM

**Fig. 4:**
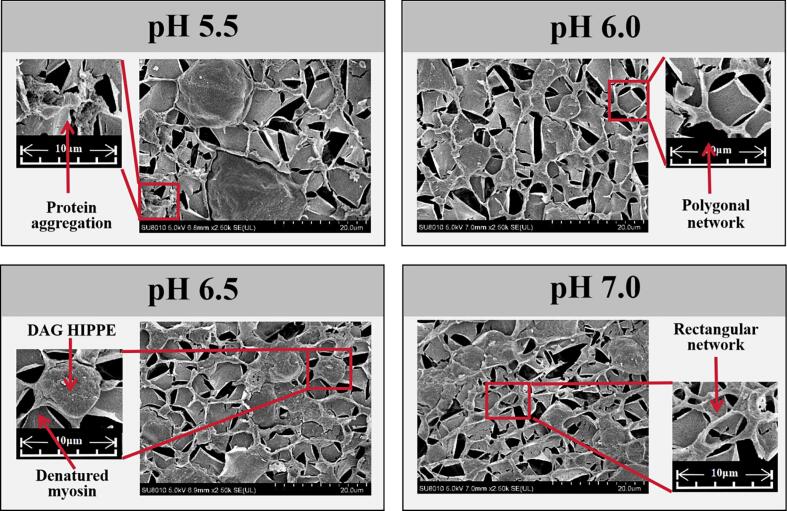
cryo-SEM images of composite gel with different pH levels (5.5, 6.0, 6.5, and 7.0).

Fig. 4 displays the cryo-SEM micrographs of the composite gels at 2500× magnification. At pH 5.5, the structure exhibited severe MP aggregation with limited cross-linking. As pH increased, enhanced electrostatic repulsion significantly reduced MP aggregation (Li et al., 2020), promoting more complete protein unfolding and cross-linking that resulted in a progressively denser gel network. The improved MP solubility (Wei et al., 2019) transformed the continuous phase morphology from distinct rod-like and spherical features to smoother, more regular surfaces. Simultaneously, the oil phase transitioned from large aggregates to finer droplets with more uniform distribution throughout the gel matrix, consistent with CLSM observations. The well-developed gel matrix combined with homogeneous oil droplet distribution contributed to the superior performance demonstrated by the composite gels, including reduced cooking loss, centrifugation loss, and thawing loss, along with enhanced liquid binding capacity evidenced by decreased free water and increased bound water content.

The gel network morphology displayed distinct variations across different pH conditions, with the continuous protein phase exhibiting a progressive stretching phenomenon. At pH 6.0, the network primarily consisted of honeycomb-like polygonal structures. The pH 6.5 samples showed mixed polygonal and elongated rectangular configurations, while the pH 7.0 gels displayed predominantly rectangular network patterns. This morphological transition from polygonal to elongated rectangular structures coincided with the slight decrease in gel strength observed at pH 6.5 and 7.0, suggesting that excessive electrostatic repulsion at higher pH may disrupt the compactness of the gel network, thereby compromising mechanical stability. Corresponding structural elongation was observed in CLSM images for both pH 6.5 and 7.0 treatments, with more pronounced stretching at the higher pH. The consistent observation of this stretching phenomenon by both CLSM and cryo-SEM techniques eliminates the possibility of sample preparation artifacts. Similar structural changes have been documented by Yang et al. (2020), while Hamm (1986) attributed such protein stretching to substantial electrostatic repulsion near neutral pH conditions. Additionally, elevated pH promotes exposure of hydrophobic amino acids in MP, facilitating non-covalent aggregate formation where thermal induction promotes longitudinal growth of aggregates through end-to-end associations (Promeyrat et al., 2010). We therefore propose that this stretching phenomenon results from strong electrostatic repulsion expanding MP structures, combined with homogenization-induced shear forces that preferentially align the proteins in specific orientations, collectively producing the observed stretching in the continuous phase.

### 3.8 Molecular forces

**Fig. 5:**
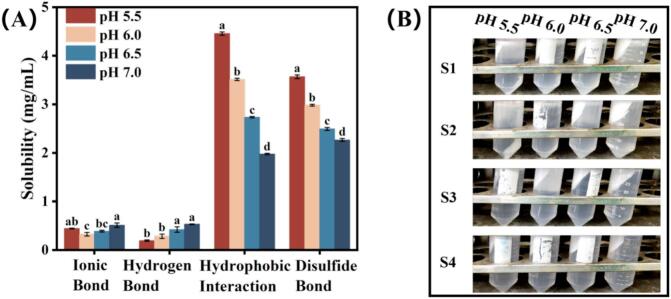
(A) Protein solubility corresponding to each chemical bond. (B) The upper layer floats and the lower layer precipitates of the composite gel with different pH levels (5.5, 6.0, 6.5, and 7.0) after treatment and centrifugation. ^a-d^ Mean values with different superscripts are statistically different (*P* < 0.05) in different pH levels. The experimental data were expressed as the mean ± standard error (SE).

The molecular forces operating within composite gels at different pH levels, including ionic bonds, hydrogen bonds, hydrophobic interactions, and disulfide bonds, are summarized in Fig. 5A. Ionic and hydrogen bonds played minor roles in structural stabilization, while hydrophobic interactions and disulfide bonds emerged as the dominant forces maintaining the gel network. Ionic bonds contributed minimally due to their susceptibility to disruption by both thermal treatment (Zhu et al., 2022) and protein-protein interactions (Yang et al., 2025). Although hydrogen bonds can collectively stabilize structures through their cumulative effect (Yang et al., 2025), individual hydrogen bonds exhibit weak thermal stability, limiting their overall contribution. The significance of hydrophobic interactions and disulfide bonds stems from MP's inherent molecular characteristics. MP contains abundant sulfur-containing amino acids capable of forming disulfide bridges under thermal induction, along with numerous hydrophobic residues (Li, Gao, et al., 2025; Yu et al., 2024). Hydrophobic interactions promoted gel formation and enhanced composite gel stability, primarily through exposure of hydrophobic groups during MP unfolding and heating (Yang et al., 2025). Concurrently, disulfide bonds formed via sulfhydryl oxidation further reinforced structural integrity (Zhang et al., 2025). Consequently, superior apparent properties in MP gels typically correlate with enhanced hydrophobic interactions and disulfide bonding (Yuan et al., 2024).

In the present study, a seemingly paradoxical reduction in both hydrophobic interactions and disulfide bond content was observed despite the enhanced stability of composite gels with increasing pH. A similar apparent contradiction has been reported in ionic strength studies on DAG HIPPE-MP composite gels, which can be attributed to the unique emulsifying properties of DAG that distinguish it from conventional edible oils (Wang, Zhou, et al., 2025). Higher pH conditions actually promote the exposure of hydrophobic groups in MP, facilitating tighter packing and stronger intermolecular associations (Gómez-Guillén et al., 1997), thereby enhancing the effectiveness of hydrophobic interactions. Simultaneously, moving away from the isoelectric point enhanced the emulsification capacity of MP for DAG (Li, Xu, et al., 2022), allowing MP to effectively envelop HIPPE droplets and form a continuous interfacial protein layer (Li, Zhao, et al., 2024). This increased interfacial adsorption of MP at higher pH aligns with the CLSM observations, which revealed a more uniform distribution of smaller HIPPE droplets within the gel matrix. During the homogenization process, exposed hydrophobic groups from MP extend into the oil phase, forming MP-DAG complexes through surface adsorption. Following centrifugation, these complexes migrate to the upper solution layer (Fig. 5B), consequently reducing the solubility of MP responsible for hydrophobic interactions in the water phase. Higher pH values promote more extensive hydrophobic group exposure, resulting in greater MP-DAG complex formation and subsequent upward migration, thereby yielding lower measured values for hydrophobic interactions. The decreased absorbance reading for S3 consequently reduces the calculated disulfide bond content (S3-S4) in the measurement system.

### 3.9 Rheological properties

#### 3.9.1 Temperature-time sweep test

**Fig. 6:**
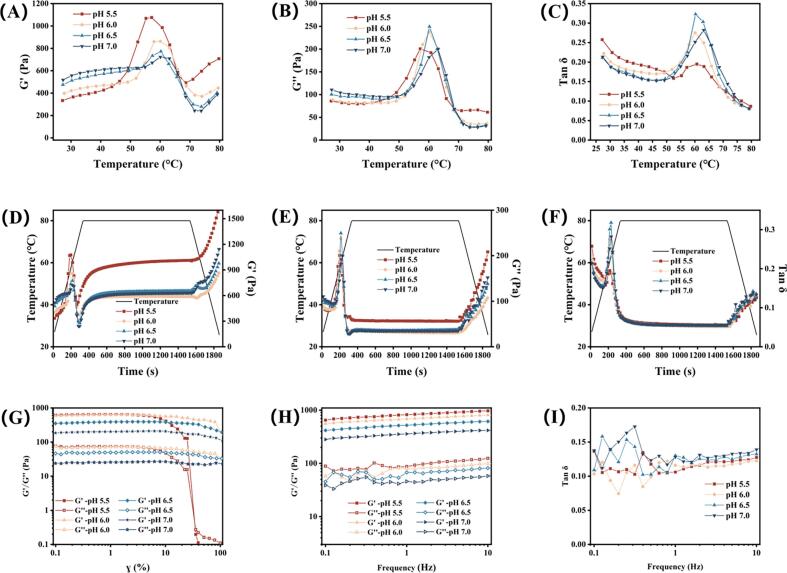
(A) Storage modulus (G'), (B) loss modulus (G") and (C) Tan δ during warming of composite gel with different pH levels (5.5, 6.0, 6.5, and 7.0). (C) G', (D) G" and (E) Tan δ during temperature-time sweep of composite gel with different pH levels (5.5, 6.0, 6.5, and 7.0). (G) Shear strain test, (H) frequency sweep test and (I) Tan δ of composite gel with different pH levels (5.5, 6.0, 6.5, and 7.0).

To simulate the thermal processing behavior of emulsified meat products, the gelation properties of the composite gels under pH influence were investigated by monitoring the temperature-dependent evolution of G' and loss G". During heating, all composite gels exhibited a consistent three-stage pattern in G' (Fig. 6A), characterized by an initial increase, followed by a decline, and a subsequent rise. The first G' increase originated from myosin head cross-linking, while the subsequent decrease resulted from the dissociation of myosin light chains. The final increase was driven by tail-domain cross-linking of myosin molecules (Wang, Zhou, et al., 2025). Although gelation is typically defined by a rising G' accompanied by decreasing Tan δ (Liu et al., 2010), our systems displayed concurrent increases in G', G" (Fig. 6B), and Tan δ (Fig. 6C) throughout the gelation process. Similar rheological patterns have been reported in κ-carrageenan-MP gels under low salt conditions (Cao et al., 2024) and in soybean oil-MP gels (Yao, Huang, et al., 2025). With increasing pH, the onset temperature of gelation shifted upward from 44 °C to 55 °C, while the gelation rate exhibited a corresponding decrease.

This behavior is attributed to protein aggregation near the isoelectric point, where proteins form large aggregates that undergo cross-linking at reduced temperatures, combined with strengthened hydrophobic interactions and disulfide bonding in acidic environments (Liu et al., 2008). Unlike the increasing G' peak trend with rising pH reported by Zhao et al. (2020) for animal fat-based DAG-MP gels, our composite gels demonstrated progressively lower G' peak values with increasing pH. This declining pattern aligns with observations in porcine myosin (Liu et al., 2008) and fish myosin systems (Liu et al., 2010). This phenomenon may be related to electrostatic repulsion. Once gelation initiated, MP at pH 5.5 underwent rapid aggregation due to limited electrostatic repulsion, forming numerous localized MP aggregates (Fig. 4). These aggregates contributed to high G' values during heating but established only weak inter-aggregate connections, resulting in a coarse, macroporous gel network. As pH increased, stronger electrostatic repulsion delayed gelation onset and shortened the effective cross-linking period, ultimately reducing the peak G' values.

During isothermal heating, the gel network underwent continuous strengthening with increasing cross-linking density. Consequently, both G' (Fig. 6D) and G" (Fig. 6E) demonstrated increasing trends throughout these stages. Tan δ decreased during isothermal heating but increased during cooling (Fig. 6F). Elevated G′ and G′′ values typically indicate higher cross-linking density, which generally corresponds to improved gel strength and water holding capacity (WHC). Paradoxically, the pH 5.5 treatment consistently showed the highest G' and G" values among all treatments, yet displayed the lowest gel strength and WHC. This apparent contradiction aligns with previous findings: Liu et al. (2010) reported that porcine myosin gels at pH 5.5 exhibited the highest G' and G" during heating while demonstrating the poorest WHC. Similarly, Westphalen et al. (2005) observed that WHC in porcine MP gels increased with pH, while G' during cooling showed higher values at lower pH.

We propose that isothermal heating and cooling promoted MP aggregation specifically in the pH 5.5 system, leading to markedly increased G' values. This phenomenon can be explained by the fact that near the isoelectric point (pH 5.5), the reduced electrostatic repulsion facilitated rapid and disordered protein aggregation upon heating. These aggregates contributed to a high elastic modulus during rheological measurement due to their dense local packing and restricted molecular mobility, yet they failed to form a well-organized, continuous three-dimensional network with effective cross-linking (Liu et al., 2008; Liu et al., 2010). Consequently, the elevated G' reflected localized aggregation rather than an integrated gel structure. The partial MP aggregation reduced the protein available for forming an interconnected gel matrix, ultimately decreasing the cross-linking degree and resulting in the inferior stability observed in the pH 5.5 gels.

#### 3.9.2 Shear strain test

Fig. 6G illustrates the variations in G' and G" under different shear strain conditions for gels at varying pH levels. Consistent with the temperature-time sweep results, higher pH values corresponded to lower G' and G" magnitudes. However, the pH 5.5 gel displayed a substantially narrower linear viscoelastic region (LVR), spanning only 0.1% to 5% strain, beyond which G′ decreased sharply. In contrast, gels at pH 6.0, 6.5, and 7.0 maintained their structural integrity up to 30% strain before showing any decline in G'. Within the LVR, the gel network remained intact, resulting in stable G' values. Once the applied strain exceeded this critical range, structural fracture occurred (Mitchell, 1980), leading to the observed decrease in G'. The significantly smaller LVR of the pH 5.5 gel indicates a more brittle three-dimensional network with inferior strain resistance. Thus, the higher G' at pH 5.5 does not indicate a stronger network or better stability. Instead, the cross linking density and structural rigidity of the MP matrix determine the strain response. Near the isoelectric point, heating promotes protein aggregation rather than a well ordered network (Liu et al., 2008; Westphalen et al., 2005), which aligns with our findings. With increasing pH, the enhanced negative charges on proteins strengthen electrostatic repulsion (Zhou et al., 2023), promoting the formation of a dense, homogeneous, and stable three-dimensional MP network through improved cross-linking. Consequently, the gels at pH 6.0, 6.5, and 7.0 exhibited broader LVRs and correspondingly superior physicochemical properties.

#### 3.9.3 Frequency sweep test

Frequency sweep results for the composite gels at different pH values are shown in Fig. 6H. Consistent with the temperature and strain sweep observations, higher pH treatments exhibited lower G' values. All samples demonstrated G' greater than G", with Tan δ values below unity (Fig. 6I), indicating predominantly elastic rather than viscous behavior (Chenite et al., 2001). Pronounced fluctuations in G" and corresponding Tan δ values occurred within the 0.1–1 Hz frequency range. We speculate that these fluctuations may result from resonance between the applied frequency and the relaxation times (τ) of structures stabilized by weak interactions, including ionic bonds, hydrogen bonds, and hydrophobic interactions. Unlike ideal polymer models characterized by single relaxation times, most polymer networks encompass multiple chain lengths and relaxation times, where polymer chain concentration determines stress magnitude and chain separation kinetics govern the relaxation spectrum (Vernerey, 2018). The distinct weak interaction profiles across treatments (Fig. 5A) correspond to variations in chain length, concentration, and separation velocity for each interaction type. The simultaneous excitation and decay of multiple relaxation processes generate the observed fluctuations in G", manifesting as multiple peaks at different frequencies within the 0.1–1 Hz range.

### 3.10 Possible reaction mechanism

**Fig. 7:**
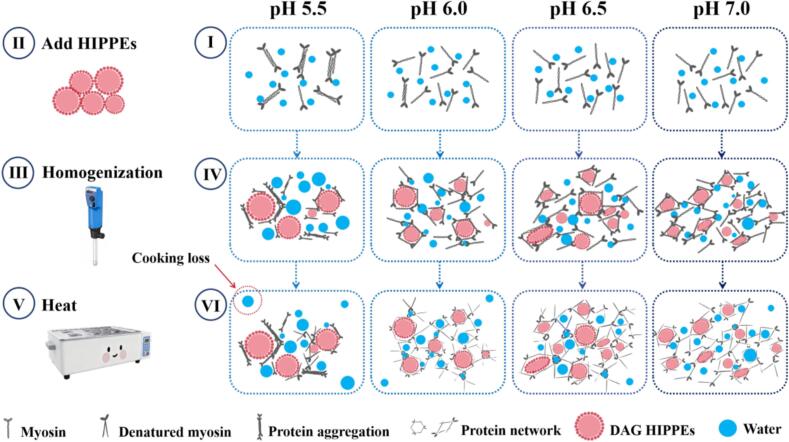
Proposed mechanisms of the effect of different pH levels (5.5, 6.0, 6.5, and 7.0) on composite gels.

This study elucidates the mechanism through which pH regulates the properties of DAG HIPPE-MP composite gels (Fig. 7). At pH 5.5, near the isoelectric point of MP, the minimal net charge substantially weakened intermolecular electrostatic repulsion, leading to marked protein aggregation. Under these conditions, thermal induction produced gel structures containing substantial MP aggregates alongside a loose and discontinuous three-dimensional network. These structural defects compromised the system's ability to effectively stabilize both water and HIPPE components. Water readily escaped through the porous gel matrix, while HIPPE droplets aggregated and distributed unevenly. The resulting phase instability further damaged the overall gel architecture.

In contrast, elevating the system pH to 6.0–7.0 markedly improved the microstructure of the composite gels. The increased net negative charge on the proteins strengthened electrostatic repulsion between MP molecules, enhancing their solubility and preventing aggregation. During heating, the unfolded MP molecules exposed additional hydrophobic groups and reactive sulfhydryl residues, leading to the formation of a dense and uniform three-dimensional network stabilized by hydrophobic interactions and disulfide bonds. This highly cross-linked matrix demonstrated a stronger capacity to immobilize water within the system.

The modified properties of both MP and SPI collectively improved the overall microstructure of the composite gels. A distinct synergistic effect emerged between the dense MP gel network and the HIPPE droplets: the uniformly distributed HIPPE droplets functioned as reinforcing fillers that strengthened the gel matrix, while the robust network effectively restricted liquid phase migration and aggregation. These microstructural improvements directly translated to enhanced macroscopic properties. As pH increased from 5.5 to 7.0, the composite gels demonstrated substantially improved stability, with significant reductions in cooking loss, centrifugation loss, and thawing loss. The gel strength achieved at pH 6.0 markedly surpassed that of the pH 5.5 samples.

Notably, further increasing the pH from 6.0 to 7.0 resulted in a slight decline in gel strength, accompanied by a morphological transition in the MP network from polygonal to elongated rectangular structures. This phenomenon likely relates to structural modifications induced by excessive electrostatic repulsion: overly fine network units combined with preferential elongation of MP structures along specific directions collectively compromised the structural stability of the gel network. These observations indicate that the DAG HIPPE-MP system exhibits an optimal pH range for performance, highlighting the balance required when optimizing complex food structures.

## 4 Conclusions

This study systematically investigated the influence of pH on the microstructure and apparent properties of DAG HIPPE-MP composite gels. At pH 5.5, the thermally induced MP network was fragile and discontinuous, containing substantial protein aggregates. This defective structure resulted in poor water retention and ineffective HIPPE stabilization, with severe droplet aggregation. Increasing the pH shifted MP from an aggregated state to an unfolded conformation, promoting the formation of a more uniform and dense three-dimensional network. Simultaneously, HIPPE aggregation was suppressed, yielding a homogeneous droplet distribution.

As pH rose from 5.5 to 7.0, the composite gels exhibited significantly reduced cooking loss, centrifugation loss, and thawing loss. The proportions of bound water and immobilized water increased, while free water decreased. Gel strength reached its maximum at pH 6.0, then slightly decreased at pH 6.5 and 7.0, suggesting that excessively strong electrostatic repulsion may compromise the gel network integrity. Analysis of molecular interactions identified hydrophobic interactions and disulfide bonds as the primary forces driving gel formation and stability.

These findings clarify the crucial role of pH in regulating the DAG HIPPE-MP composite system and provide a theoretical foundation for optimizing DAG HIPPE-based meat products through precise pH control. It is important to note that this finding was obtained in a simplified model system, and the effects under more acidic or alkaline conditions were not explored. In real meat product applications, the presence of additional components and different processing conditions may influence the system's response to pH, warranting further investigation under more complex, application-relevant conditions.

## CRediT authorship contribution statement

**Ziyi Wang:** Writing – original draft, Visualization, Investigation, Formal analysis, Conceptualization. **Yuxin Huang:** Software, Investigation, Formal analysis. **Yafei Zhou:** Software, Investigation. **Chao Zhang:** Investigation, Formal analysis. **Qian Liu:** Visualization, Investigation. **Qian Chen:** Investigation, Formal analysis. **Simin Zheng:** Investigation, Formal analysis. **Haotian Liu:** Writing – review & editing, Funding acquisition. **Baohua Kong:** Supervision, Software, Project administration, Investigation.

## Declaration of competing interest

The authors declare that they have no known competing financial interests or personal relationships that could have appeared to influence the work reported in this paper.

## Data Availability

Data will be made available on request.
